# Estimating Contraceptive Prevalence Using Logistics Data for Short-Acting Methods: Analysis Across 30 Countries

**DOI:** 10.9745/GHSP-D-15-00116

**Published:** 2015-09-10

**Authors:** Marc Cunningham, Ariella Bock, Niquelle Brown, Suzy Sacher, Benjamin Hatch, Andrew Inglis, Dana Aronovich

**Affiliations:** ^a^​ John Snow, Inc., Arlington, VA, USA; ^b^​ University of Southern California, Los Angeles, CA, USA; ^c^​ JSI Research & Training Institute, Inc., Arlington, VA, USA

## Abstract

**Background:**

Contraceptive prevalence rate (CPR) is a vital indicator used by country governments, international donors, and other stakeholders for measuring progress in family planning programs against country targets and global initiatives as well as for estimating health outcomes. Because of the need for more frequent CPR estimates than population-based surveys currently provide, alternative approaches for estimating CPRs are being explored, including using contraceptive logistics data.

**Methods:**

Using data from the Demographic and Health Surveys (DHS) in 30 countries, population data from the United States Census Bureau International Database, and logistics data from the Procurement Planning and Monitoring Report (PPMR) and the Pipeline Monitoring and Procurement Planning System (PipeLine), we developed and evaluated 3 models to generate country-level, public-sector contraceptive prevalence estimates for injectable contraceptives, oral contraceptives, and male condoms. Models included: direct estimation through existing couple-years of protection (CYP) conversion factors, bivariate linear regression, and multivariate linear regression. Model evaluation consisted of comparing the referent DHS prevalence rates for each short-acting method with the model-generated prevalence rate using multiple metrics, including mean absolute error and proportion of countries where the modeled prevalence rate for each method was within 1, 2, or 5 percentage points of the DHS referent value.

**Results:**

For the methods studied, family planning use estimates from public-sector logistics data were correlated with those from the DHS, validating the quality and accuracy of current public-sector logistics data. Logistics data for oral and injectable contraceptives were significantly associated (*P*<.05) with the referent DHS values for both bivariate and multivariate models. For condoms, however, that association was only significant for the bivariate model. With the exception of the CYP-based model for condoms, models were able to estimate public-sector prevalence rates for each short-acting method to within 2 percentage points in at least 85% of countries.

**Conclusions:**

Public-sector contraceptive logistics data are strongly correlated with public-sector prevalence rates for short-acting methods, demonstrating the quality of current logistics data and their ability to provide relatively accurate prevalence estimates. The models provide a starting point for generating interim estimates of contraceptive use when timely survey data are unavailable. All models except the condoms CYP model performed well; the regression models were most accurate but the CYP model offers the simplest calculation method. Future work extending the research to other modern methods, relating subnational logistics data with prevalence rates, and tracking that relationship over time is needed.

## Abstract (precis)

Three models showed strong correlation between public-sector logistics data for injectables, oral contraceptives, and condoms and their prevalence rates, demonstrating that current logistics data can provide useful prevalence estimates when timely survey data are unavailable.

## BACKGROUND

Access to contraceptives and reproductive choice are considered basic human rights.^1^^,^^2^ Increased access to and use of contraceptives for family planning have been linked to improved economic growth and decreased maternal and child mortality.^3^^-^^5^ However, more than 220 million women in developing countries want to space or limit their pregnancies but are not using a modern method of contraception.^6^ In recent years, there have been concerted program efforts to increase the availability and use of contraceptives through demand generation, improved service delivery, and supply chain strengthening.

Monitoring contraceptive prevalence rates (CPRs) is key for maternal and reproductive health programs to identify areas in need of increased focus and to guide resource allocations from national governments, donors, and civil society. CPR estimates have historically been collected every 5 years through population-based surveys, such as the Demographic and Health Surveys (DHS).^7^ However, updates every 5 years are proving to be insufficient for informing optimal allocation of limited resources; up-to-date estimates, obtained at shorter intervals, are needed to allow for adequate tracking.^8^ This is particularly true given that there is less than 5 years left to meet the Family Planning 2020 (FP2020) partnership goal of enabling 120 million more women and girls to use modern contraceptives by 2020.^6^

Population-based surveys are usually conducted every 5 years, but CPR updates are often needed more frequently.

Because of the need for more frequent CPR estimates than the DHS series currently provides, alternative approaches for estimating CPRs are being explored. These include continuous or annual population-based surveys,^9^^,^^10^ extrapolations from previous surveys,^11^ and using contraceptive logistics data.^11^^-^^13^ However, continuous and annual population-based surveys require substantial resources that countries might not be prepared to invest, while extrapolations from previous surveys provide no information about recent changes to CPRs if these countries do not follow historical trends—precisely what we would expect in the context of the intensified focus on dramatically increasing CPRs by 2020.

Since the late 1960s, family planning programs have used contraceptive service delivery data to calculate couple-years of protection (CYP) to estimate program impact.^14^^,^^15^ CYP conversion factors are applied to contraceptive service delivery data on commodities distributed in order to estimate client use within programs.^16^^,^^17^ By combining client use with population data, programs can estimate their contribution to overall country CPR.^16^^,^^18^ Information on client use can typically be found in a country’s routine health information system or in its logistics management information system (LMIS).

Logistics data are collected primarily to help manage stock levels and determine resupply quantities. Three essential logistics data points reported by health facilities and warehouses include^19^:

- The quantities of products in stock, referred to as stock on hand
- The quantities of products dispensed to patients or clients or issued to a lower-level warehouse or health facility during the reporting period
- Any losses or adjustments to the stock balance due to damage, expiries, or transfers between facilities

Of supply chain management data collected for health products, data capturing the quantities of commodities dispensed to clients are considered the best approximation of client use. Other approximations of client use are based on quantities of commodities issued by facility storerooms to dispensaries, quantities of commodities issued by warehouses and stores to service delivery points, and forecasted dispensed-to-user data.^19^ Where accurate contraceptive logistics data reflect client use, it is reasonable to assume that such data can provide a low-cost alternative to surveys for regularly estimating CPRs.

Accurate contraceptive logistics data may provide a low-cost alternative to surveys for regularly estimating CPR.

A recent study in Rwanda found a strong positive correlation between the quantities of public-sector injectable contraceptives dispensed to users and the DHS-reported, public-sector injectable prevalence rate, leading to the conclusion that Rwanda’s public-sector LMIS appeared to be accurately capturing logistics data and that logistics data reflect client use.^12^ With the expansion of national-level LMISs as part of efforts to strengthen government supply chains, such logistics data are more readily accessible for other countries as well. Despite this accessibility, there have been limited examples of using logistics data to estimate national CPR.

To help address this gap, this study examines the relationship between public-sector prevalence levels of short-acting methods and contraceptive logistics distribution data from 30 countries. We argue that logistics data can be used to generate prevalence estimates for short-acting contraceptives when up-to-date survey data are unavailable, and we evaluate 3 models for doing so.

## METHODS

We calculated country-level, public-sector prevalence estimates for short-acting contraceptives (injectable contraceptives, oral contraceptives, and male condoms) for all women of reproductive age (WRA), i.e., 15 to 49 years of age, from DHS datasets. Based on a combination of DHS, population data, and logistics data, for each of the short-acting methods we developed 3 models that could be used to generate country-level, public-sector prevalence estimates. Models included: direct estimation using CYP conversion factors, bivariate linear regression, and multivariate linear regression.

To model prevalence estimates using CYP conversion factors, we converted logistics data on the number of injectable contraceptives, oral contraceptives, and male condoms distributed to CYP, using the conversion factors recognized by the United States Agency for International Development (USAID), and then divided by the number of WRA for each country.^20^ For the regression models, we likewise examined each method separately, first using a bivariate model and then adjusting for a previous national prevalence observation. We evaluated each model for each method by examining how accurately the model-generated prevalence values matched the referent DHS values.

This research was deemed to be exempt from institutional review board (IRB) approval by the John Snow IRB.

### Data, Indicators, and Country Inclusion

We obtained public-sector logistics data from the Procurement Planning and Monitoring Report (PPMR) and the Pipeline Monitoring and Procurement Planning System (PipeLine). PPMR data track information on contraceptive stock levels and on product distribution. Such data enable the Coordinated Assistance for Reproductive Health Supplies group, convened by the Reproductive Health Supplies Coalition, to better address supply issues.^21^ PipeLine is used by program managers to plan procurement based on estimated future needs and to subsequently track shipments and product distribution.^22^ More than 50 countries currently report or have reported since 1997 public-sector contraceptive logistics data via PPMR or PipeLine. The USAID | DELIVER PROJECT, which receives the data and manages both systems on behalf of countries and donors, provided the data for this analysis.

Country programs report their logistics distribution data by different metrics, including forecast data (amount expected to be dispensed to users during the period); issues data (amount distributed to health facility dispensaries, health facility stores, or lower-level warehouses, from health facility stores, district stores, or other higher-level warehouses); and dispensed-to-user data (actual amount provided to clients). While dispensed-to-user data are considered to be the “gold standard” for logistics data, forecast and issues data are recognized as acceptable proxies for tracking distribution and planning purposes in the absence of dispensed-to-user data.

Dispensed-to-user data are considered the “gold standard” for logistics data, but forecast and issues data are acceptable proxies.

Public-sector, country-level prevalence values for short-acting methods were obtained from the DHS program for 30 countries for which there were DHS data for time periods overlapping the logistics data (Table 1). These DHS data were used as dependent variables in the regression models and as the reference national contraceptive use values for evaluation of all 3 models.

**TABLE 1:** Countries Included in the Analysis, Data Sources, and Periods of Analysis

Country	Country Code	DHS Collection Dates	Previous DHS	Contraceptive Logistics Data Source	Logistics Data Dates
Bangladesh	BD	7/2011–12/2011	2007	PPMR	6/2011–12/2011
Bolivia	BO	8/2003–1/2004	1998	PipeLine	7/2003–2/2004
Burkina Faso	BF	5/2010–12/2010	2003	PipeLine	4/2010–1/2011
Cameroon	CM	2/2004–9/2004	1998	PipeLine	1/2004–10/2004
Côte d'Ivoire	CI	12/2011–5/2012	1998–99	PPMR	11/2011–6/2012
Ethiopia	ET	4/2011–9/2011	2005	PPMR	1/2011–1/2012
Ghana	GH	9/2008–11/2008	2003	PipeLine	8/2008–12/2008
Guinea	GN	2/2005–6/2005	1999	PipeLine	1/2005–7/2005
Haiti	HT	1/2012–6/2012	2007	PPMR	3/2011–9/2012
Honduras	HN	10/2005–5/2006	--	PipeLine	9/2005–6/2006
Jordan	JO	7/2002–10/2002	1997	PipeLine	6/2002–11/2002
Kenya	KE	11/2008–3/2009	2003	PPMR	10/2008–5/2009
Liberia	LR	12/2006–4/2007	--	PipeLine	11/2006–5/2007
Madagascar	MD	11/2008–7/2009	2005	PipeLine	10/2008–8/2009
Malawi	MW	6/2010–10/2010	2006	PPMR	5/2010–11/2010
Mali	ML	4/2006–12/2006	2001	PipeLine	3/2006–12/2006
Mozambique	MZ	5/2011–12/2011	2005	PPMR	4/2011–6/2011
Nepal	NP	1/2011–6/2011	2006	PPMR	1/2011–7/2011
Nicaragua	NC	9/2001–12/2001	1998–99	PipeLine	8/2001–1/2002
Niger	NI	2/2012–7/2012	2006	PPMR	6/2013
Nigeria	NG	6/2008–11/2008	2003	PipeLine	7/2008–12/2008
Pakistan	PK	10/2012–4/2013	2006–07	PPMR	12/2012–3/2012
Philippines	PH	6/2003–9/2003	1998	PipeLine	2/2003–11/2003
Rwanda	RW	9/2010–4/2011	2009	PPMR	6/2010–5/2011
Senegal	SN	10/2010–5/2011	2005	PPMR	7/2010–7/2011
Tanzania	TZ	12/2009–5/2010	2006	PPMR	9/2009–6/2010
Togo	TG	2/1998–5/1998	1988	PipeLine	1/1998–6/1998
Uganda	UG	6/2011–12/2011	2006	PPMR	1/2011–12/2011
Zambia	ZM	4/2007–10/2007	2003	PipeLine	3/2007–11/2007
Zimbabwe	ZW	9/2010–3/2011	2007	PPMR	6/2010–6/2011

Abbreviations: DHS, Demographic and Health Surveys; PipeLine, Pipeline Monitoring and Procurement Planning System; PPMR, Procurement Planning and Monitoring Report.

For each country, the number of WRA was obtained from the United States Census Bureau International Database based on the year of the DHS and logistics data.^23^ When the DHS data spanned 2 calendar years, we used an average of the midyear totals of the numbers of WRA.

### Contraceptive Method and Sector Inclusion

We sought to evaluate how well logistics data could be used to estimate prevalence rates for individual methods. We focused on the 3 most commonly used types of short-acting contraceptive methods—oral contraceptives, injectable contraceptives, and male condoms—assuming the relationship between commodities distributed and national prevalence for these commodities in a specific year is likely stronger than for long-acting methods, for which efficacy lasts over multiple years. Long-acting methods were also excluded due to the lack of countries with overlapping logistics and DHS data (n<20).

DHS collects data on both private and public-sector CPR, but PPMR and PipeLine primarily collect public-sector distribution data. Therefore, we limited our analysis to public-sector prevalence of the short-acting methods.

### Calculating Average Distribution From Logistics Data

For each country, we sought to determine the amount of each commodity distributed per 100 WRA in a given time period. For male condoms and oral contraceptives, which are often distributed to clients at least once per month, we calculated average monthly distribution (AMD). For injectable contraceptives, we calculated average quarterly distribution (AQD) because the primary form of injectable contraceptive used in the countries included in our analysis, depot medroxyprogesterone acetate (DMPA), is administered to clients once every 3 months. We converted any distribution data for monthly injectable contraceptives into the equivalent for quarterly injectables to aggregate the data and calculate injectable AQD. Oral contraceptive AMD was calculated from aggregated progestin-only and combined oral contraceptive logistics data because the DHS does not differentiate between these 2 oral contraceptives when reporting results.

AMD and AQD were combined with population data to calculate the average amount of each commodity distributed per 100 WRA in order to provide a population-standardized estimate of distribution of each method in each country based on the logistics data available. As our outcome indicator—public-sector contraceptive prevalence for short-acting methods—is population-based, it was important that we had population-based dependent variables.

### Analysis

For each country, we examined the CPR for modern methods (mCPR) as well as the prevalence rate for each short-acting method and the public-sector market share from the DHS in order to assess both the contributions of the short-acting methods and the public-sector market for these methods to overall modern contraceptive use. As public-sector, short-acting methods account for the vast majority of mCPR in most countries examined, quantifying their relative contributions provides insight into how reflective our models might be of overall mCPR. We then created 3 models, using the logistics data, to estimate country-level, public-sector prevalence rates for each short-acting method and evaluated the performance of each model.

For the first model, we estimated the public-sector prevalence rate for each method using CYP conversion factors: we converted logistics data on injectable contraceptives, oral contraceptives, and male condoms into CYP and then divided the method-specific CYP by the number of WRA for each country to estimate the prevalence rates for each short-acting method.

For the second and third models, we applied bivariate and multivariate linear regressions, respectively. For each contraceptive method, the bivariate model examined the association between public-sector AMD or AQD per 100 WRA (independent variable) and the public-sector prevalence rate for that method from the most recent DHS (dependent variable). The multivariate model examined the same association, adjusting for previous public-sector prevalence rate for each method (from the prior DHS), a potential confounder given that current contraceptive requirements and current use are both influenced by historic rates of contraceptive use. To meet the assumptions for linear regressions, due to a skewed dataset, we applied a natural log transformation to both the prevalence rate for each short-acting method (dependent variable) and AMD or AQD per 100 WRA (independent variable) in the bivariate and multivariate models.^24^

Thus, the equation for the bivariate model was: 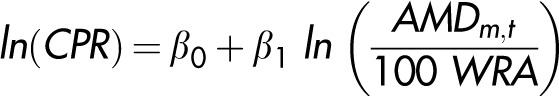
where β_1_ is the coefficient for the public-sector commodity distribution data as measured by the logistics data for the corresponding time period, β_0_ is the slope intercept, and (AQD or AMD)_m,t_ per 100 WRA is the LMIS-based distribution data of oral contraceptives, injectable contraceptives, or condoms for method *m* in year *t* divided by 100 WRA to simulate the prevalence rate for each method.

The multivariate model was built upon the bivariate model by adding 1 covariate—the term CPR_m,t−i_, which is the DHS-based estimate of prevalence of use for method *m* in year *t−i*, where *i* is the interval since the last survey. Thus, the equation was:

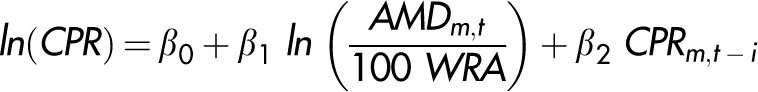


Using these models, we calculated model-generated, public-sector prevalence estimates by method for each of the 30 countries based on country logistics data (AMD or AQD/100 WRA) for the CYP, bivariate, and multivariate models. We summed the model-generated values for condoms, injectable contraception, and oral contraceptives to create multivariate, bivariate, and CYP “combined public-sector, short-acting methods” models. The results these models provide for each country can be interpreted as a “best estimate” for that country’s prevalence rates for each short-acting method.

Oral contraceptive data from 2 countries (Bangladesh and Zimbabwe) were dropped from analysis due to data analysis concerns. These 2 countries had substantially higher AMD/100 WRA values for oral contraception (>20/100 WRA) than the other 28 countries, and therefore were influential outliers with undue weight in the regression models. Additionally, 2 other countries (Madagascar and Niger) did not have logistics data on condoms and 1 country (Uganda) did not have data on oral contraceptives, so these countries were excluded from the analyses for the respective methods.

All analyses were performed using Stata version 12 (College Station, TX).

### Evaluation of Models

We evaluated the bivariate, multivariate, and CYP-based models by comparing the referent DHS prevalence rates for each short-acting method with the model-generated prevalence rates for each method using multiple metrics, including mean absolute error (MAE) and proportion of countries where the modeled prevalence rate by method was within 1, 2, or 5 percentage points of the referent prevalence rate. MAE is a standard calculation for model comparisons. To generate the MAE, we first subtracted model-generated prevalence rate from the referent prevalence rate. The absolute value of this difference, per country, is the model absolute error value. The MAE is the average of these values. For our models, high MAE values correspond to high error values (less accurate models). We chose 1, 2, and 5 percentage-point cutoffs as levels of precision that would have programmatic value.

## RESULTS

### Method Mix and Market Share

While most of the CPR in countries was attributable to oral contraceptives, condoms, and injectables, method mix varied substantially in the countries examined (Table 2). In Bangladesh and Zimbabwe, oral contraceptive use at the time of the DHS was high (about 27% of WRA). In Malawi, injectable contraceptives were used by 19.2% of WRA, while in Cameroon and Pakistan, male condoms were the most prevalent method (9.7% and 8.8% of WRA, respectively).

**TABLE 2:** Modern Contraceptive Prevalence Rate (mCPR), Prevalence Rates of Short-Acting Methods, and Public-Sector Market Share, by Country, From DHS

		Prevalence (%)			Public-Sector Prevalence (%)			Public-Sector Market Share (%)		
Country	mCPR (%)	OC	IC	MC	OC	IC	MC	OC	IC	MC
Bangladesh	52.1	27.2	11.2	5.5	12.2	7.4	0.9	45.0	66.5	16.8
Bolivia	23.7	2.5	5.3	3.1	0.8	4.0	0.2	31.5	74.5	7.5
Burkina Faso	14.3	2.8	5.1	3.1	2.3	5.0	0.3	83.4	97.3	8.9
Cameroon	13.5	1.3	1.1	9.7	0.6	0.8	0.6	49.3	75.0	6.4
Côte d'Ivoire	13.9	6.1	1.9	5.0	1.4	1.7	0.2	23.4	89.2	4.2
Ethiopia	18.7	1.5	14.0	0.3	1.0	12.1	0.0	67.3	86.3	11.7
Ghana	13.5	3.6	4.2	3.6	0.5	3.7	0.1	12.8	87.0	2.7
Guinea	6.8	1.6	1.1	2.5	0.7	0.9	0.2	42.0	86.2	7.5
Haiti	21.6	1.7	11.7	5.8	0.4	5.5	0.7	22.4	46.6	11.2
Honduras	37.7	7.1	8.6	2.3	2.0	6.2	0.6	28.4	72.2	24.2
Jordan	41.2	7.5	0.9	3.4	2.7	0.4	1.3	36.5	46.7	37.3
Kenya	28.0	4.7	14.8	2.6	2.0	9.7	0.5	42.6	65.3	20.5
Liberia	11.7	3.8	3.7	3.5	2.2	2.6	1.4	56.8	69.1	40.9
Madagascar	23.0	4.8	14.1	1.0	2.8	11.7	0.0	57.3	82.9	4.9
Malawi	32.6	1.9	19.2	2.7	1.6	16.2	1.2	81.8	84.4	46.1
Mali	6.2	2.6	2.2	0.5	1.0	1.7	0.0	36.8	76.7	3.7
Mozambique	12.1	4.3	4.3	2.9	3.7	4.1	1.0	86.2	95.4	34.8
Nepal	33.2	3.2	7.0	3.3	1.6	4.8	1.1	50.9	69	32.3
Nicaragua	43.9	9.0	9.1	2.2	5.3	6.8	0.8	59.4	74.3	35.8
Niger	11.0	5.0	1.9	0.1	4.1	1.8	0.1	82.9	94.4	69.2
Nigeria	11.1	1.6	2.0	4.7	0.3	1.1	0.2	19.0	54.7	4.0
Pakistan	26.1	1.6	2.8	8.8	0.8	1.6	1.6	47.7	56.5	17.8
Philippines	23.5	8.4	2.0	1.2	4.8	1.9	0.3	56.6	92.5	27.4
Rwanda	25.2	3.9	14.6	1.8	3.7	14.2	0.9	94.2	97.1	51.4
Senegal	8.9	2.9	3.7	0.6	2.4	3.5	0.1	82.4	94.8	20.7
Tanzania	23.6	5.1	8.5	4.2	3.7	6.8	0.7	73.5	80.0	17.0
Togo	7.9	1.1	1.7	3.4	0.4	1.6	0.5	39.5	91.6	15.0
Uganda	20.7	2.1	10.7	3.2	1.0	4.2	0.9	45.7	39.1	28.6
Zambia	24.6	7.4	6.2	5.0	4.5	5.7	2.6	61.3	92.1	51.7
Zimbabwe	40.5	27.3	6.1	3.5	20.2	5.4	1.6	73.8	88.4	45.9

Abbreviations: CPR, contraceptive prevalence rate; DHS, Demographic and Health Surveys; IC, injectable contraceptives; MC, male condoms; mCPR, CPR for modern methods; OC, oral contraceptives.

In the countries in this study, most women using any type of contraception received their method from a public-sector facility, e.g., a government clinic or hospital (Table 2). However, the source of the method varied when analyzing market share by individual product—the majority of women using injectable contraceptives reported receiving the method from public sources, while those using condoms were more likely to get them from nonpublic sources such as NGOs, private pharmacies, or other private stores. With oral contraceptives, there was more variation between countries in public versus nonpublic sources of supply.

The role of the public sector varied between survey years (data not shown). For instance, in Bolivia, although the oral contraceptive prevalence rate remained constant at approximately 3% over the 5-year period between surveys, its public-sector market share increased from 20% in 1998 to 32% in 2003–2004. On the other hand, in Nepal, where the prevalence rate for injectable contraceptives declined slightly from 8% to 7%, the public market share for injectable contraception decreased more substantially from 82% in 2006 to 69% in 2011.

### CYP Model and Regression Models

For each of the 3 models (bivariate, multivariate, and CYP), we used logistics data to create model-generated prevalence estimates for each contraceptive method and country (see Supplemental Table 1 for injectable values, Supplemental Table 2 for oral contraceptive values, Supplemental Table 3 for male condom values, and Supplemental Table 4 for values for all short-acting methods combined).

The significant *P* values (*P*≤.001) in the bivariate regression models indicate a strong linear relationship on the natural log scale between the public-sector prevalence rates and logistics data for all short-acting methods (Table 3). The proportion of variance explained varied by method—the model R^2^ term was highest for injectable contraceptives (0.90), followed by oral contraceptives (0.48) and condoms (0.28). The bivariate model β_1_ coefficients were 0.72, 0.45, and 0.44 for injectable contraceptives, oral contraceptives, and condoms, respectively. As the regressions were conducted on a log-log scale, these coefficients can be interpreted as the **marginal percent increase** in the prevalence rate of a method given a 1% rise in AMD or AQD/100 WRA. Thus, based on the β_1_ coefficient for injectable contraceptives, a marginal increase of 10% in the AQD of injectable contraceptives per 100 WRA would be associated with a corresponding marginal increase of 7.2% in the prevalence rate for injectables.

**TABLE 3:** Association Between Referent Public-Sector Prevalence Rates and Average Monthly or Quarterly Logistics Distribution Data, by Contraceptive Type and Model Type

Model and Contraceptive Type	N	β_0_	β_1_	β_2_	R^2^-adj
Bivariate Model					
Injectable contraceptives	30	−4.11	0.72***	NA	0.90
Oral contraceptives^a^	27	−4.46	0.45***	NA	0.48
Male condoms	28	−6.49	0.44***	NA	0.28
Multivariate Model					
Injectable contraceptives	28	−4.21	0.62***	5.7	0.91
Oral contraceptives^a^	25	−4.97	0.23*	34.93***	0.72
Male condoms	26	−6.66	0.19	171.93***	0.48

^a^​ The analysis was restricted to countries with <20 average monthly distribution per 100 women of reproductive age.

* *P*<.05, ** *P*<.01, *** *P*<.001.

Public-sector prevalence rates for injectables, oral contraceptives, and condoms were significantly associated with logistics data in the bivariate model.

After controlling for the prevalence rates of each short-acting method from previous surveys in the multivariate model, the association between the model-generated, public-sector prevalence rates and the referent DHS prevalence rates remained significant for both injectables (β_1_ = 0.62, *P*≤.001, R^2^ = 0.91) and oral contraceptives (β_1_ = 0.23, *P*≤.05, R^2^ = 0.72) but lost its significance with male condoms (β_1_ = 0.19, *P*>.1, R^2^ = 0.48) (Table 3). Similar to the bivariate model, multivariate model β_1_ coefficients can be interpreted as the **marginal percent increase** in the prevalence rate of a method given a 1% rise in AMD or AQD/100 WRA.

The model equations can also be used to create model-generated estimates of a country’s public-sector prevalence rate for each method. For the injectables bivariate model, the slope intercept (β_0_) is -4.11, and β_1_ is 0.72 (Table 3). Taking Tanzania as an example with its AQD/100 WRA of 10.1 in 2009–10, the bivariate model generated a public-sector injectables prevalence rate estimate of 8.6% (see Supplemental Table 1). The multivariate model yielded an injectables prevalence rate estimate of 8.7%, and the CYP model an estimate of 10.1%. These can be compared with the referent DHS value of 6.8%.

**FIGURE 1:**
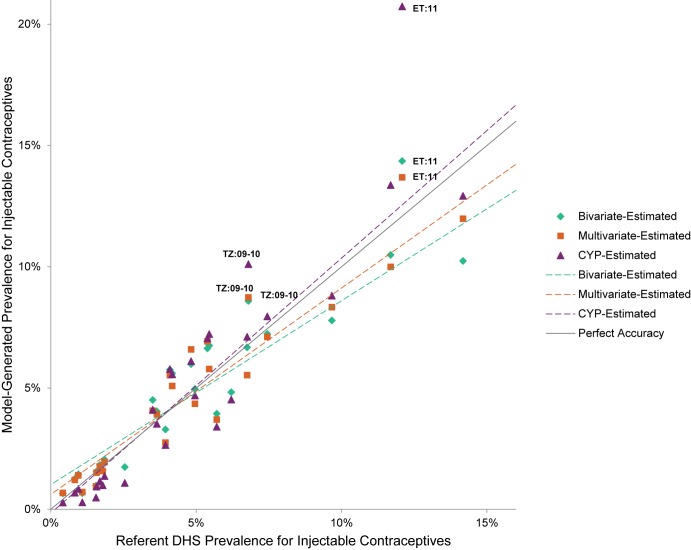
Public-Sector Injectables Prevalence Rate Estimates

**FIGURE 2:**
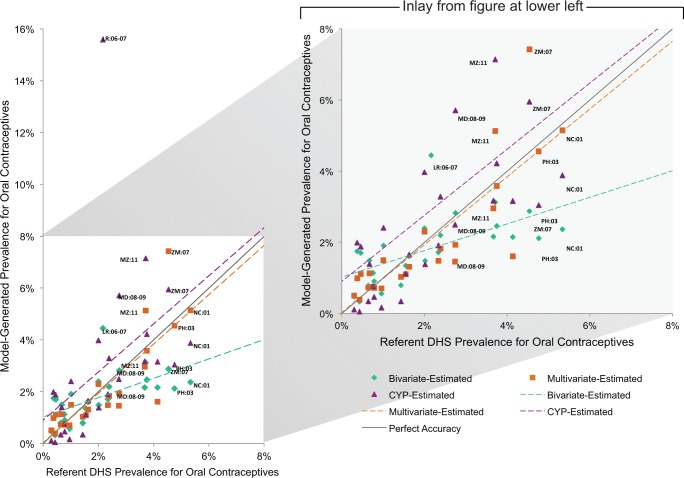
Public-Sector Oral Contraceptive Prevalence Rate Estimates

**FIGURE 3:**
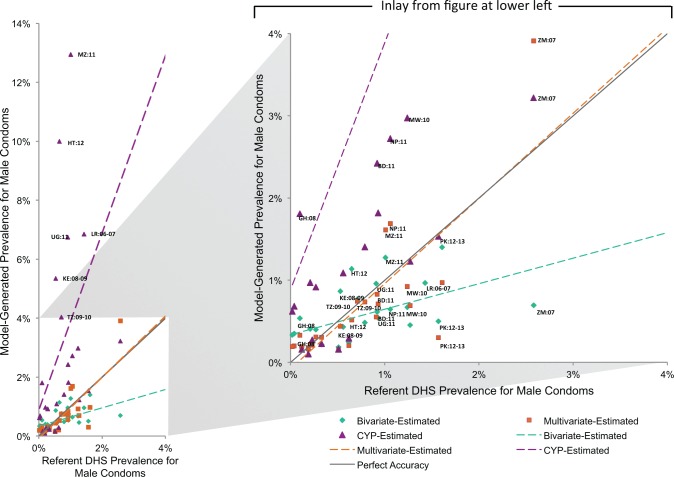
Public-Sector Male Condom Prevalence Rate Estimates

**FIGURE 4:**
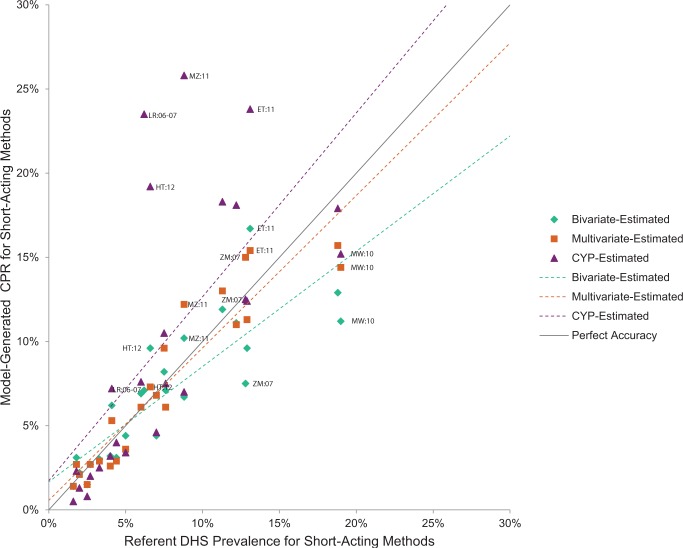
CPR Estimates for Public-Sector Short-Acting Methods

The Figures compare model-generated prevalence rate values with those from the referent DHS (Figure 1 for injectables, Figure 2 for oral contraceptives, Figure 3 for male condoms, and Figure 4 for all short-acting methods combined). In cases where the model-generated prevalence values match the referent DHS values exactly (i.e., data points falling on the gray line with a slope of 1), the model provides a completely accurate estimation. The data points present a comparison of the model-generated and referent prevalence rates: green diamond for the bivariate model; orange square for the multivariate model; and purple triangle for the CYP model. Model-generated public-sector prevalence values for countries above the line are overestimated while those below are underestimated. Again, using Tanzania injectable contraceptives as an example, the bivariate model overestimates the “true” injectables prevalence rate by 1.8 percentage points; the multivariate model by 1.9 percentage points; and the CYP model by 3.3 percentage points. For context, we note that the 2010 Tanzania DHS’s margin of error for the public-sector injectables rate is 0.8 percentage points.

In general, the model-generated estimates for injectable contraception (across all models) were more accurate than for other methods—the majority of data points fell closer to the gray line, and the overall spread of the points were neither overestimates or underestimates (as seen by the slopes of the model lines, which were closer to that of the gray line) (Figure 1). For oral contraceptives, the bivariate model fared least well, differentially underestimating the oral contraceptives prevalence rate for higher (≥4%) values (Figure 2). For condoms, there were 6 countries that fell far above the gray line for the CYP model, indicating substantial overestimation (Figure 3). Similarly, the countries that fell below the gray line for the bivariate model reflect its underestimation for higher values.

Model-generated prevalence estimates were generally more accurate for injectables than for other methods.

### Model Evaluation

As mentioned earlier, we evaluated each model by comparing model-generated public-sector prevalence rates for each short-acting method with corresponding DHS values using MAE and by examining the proportion of countries where the model-estimated prevalence rate was within 1, 2, or 5 percentage points of the DHS referent value for the method. We also evaluated the maximum absolute error, which shows the highest model error for any country for a specific method, and the median error—a measure which is less sensitive to outliers. Models with lower mean, median, and maximum error perform better than those with higher values.

Models performed well at estimating public-sector prevalence rates from logistics data. The MAE for the individual method models ranged from 0.3 to 2.4 percentage points (Table 4). However, the regression models performed better than the CYP-based estimation model, as seen by the fact that the regression models’ error values were lower across all contraceptive methods. With the exception of the CYP condoms model, all method-specific models were able to accurately estimate, to within 2 percentage points, a country’s public-sector prevalence rate for each method for at least 85% of the countries in the analysis (Table 4, the “2 Percentage Points” column). Models fared less well in estimating accuracy to within 1 percentage point.

**TABLE 4:** Evaluation of Model Accuracy and Precision

	Difference Between Model Estimates and DHS Referent Values			Proportion of Model-Estimated Values Within 1, 2, and 5 Percentage Points of the DHS Value		
Model	Maximum Absolute Error (%)	Mean Absolute Error (MAE) (%)	Median Absolute Error (%)	1 Percentage Point (%)	2 Percentage Points (%)	5 Percentage Points (%)
Injectables						
Multivariate	3.8	1.0	0.6	57	89	100
Bivariate	7.0	1.1	0.7	57	90	97
CYP	8.6	1.4	0.8	54	86	93
Oral Contraceptives						
Multivariate	2.9	0.6	0.4	84	92	100
Bivariate	3.0	0.9	0.6	67	89	100
CYP	3.4	1.0	0.8	60	92	100
Condoms						
Multivariate	1.3	0.3	0.2	92	100	100
Bivariate	1.9	0.4	0.3	93	100	100
CYP	14.4	2.4	0.6	62	77	85
All Short-Acting Methods						
Multivariate	4.6	1.4	1.3	35	74	100
Bivariate	7.8	1.9	1.2	40	64	88
CYP	17.0	3.4	1.5	43	61	78

While all 3 models generally performed well at estimating contraceptive prevalence, the regression models provided more accurate estimates than the CYP model.

For the combined short-acting methods model, on average (based on MAE), we were able to estimate countries’ public-sector CPRs attributable to these short-acting methods to within 1.4 percentage points using the multivariate model and to within 1.9 percentage points using the bivariate model (Table 4).

**APPENDIX FIGURE 1.**
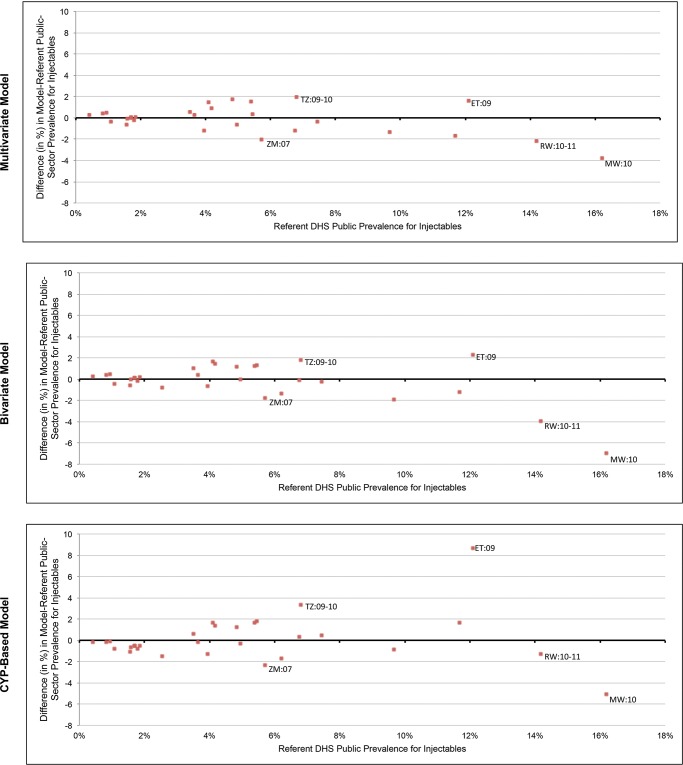
Difference Between Model-Generated and Referent DHS Public Injectables Prevalence Rate

**APPENDIX FIGURE 2.**
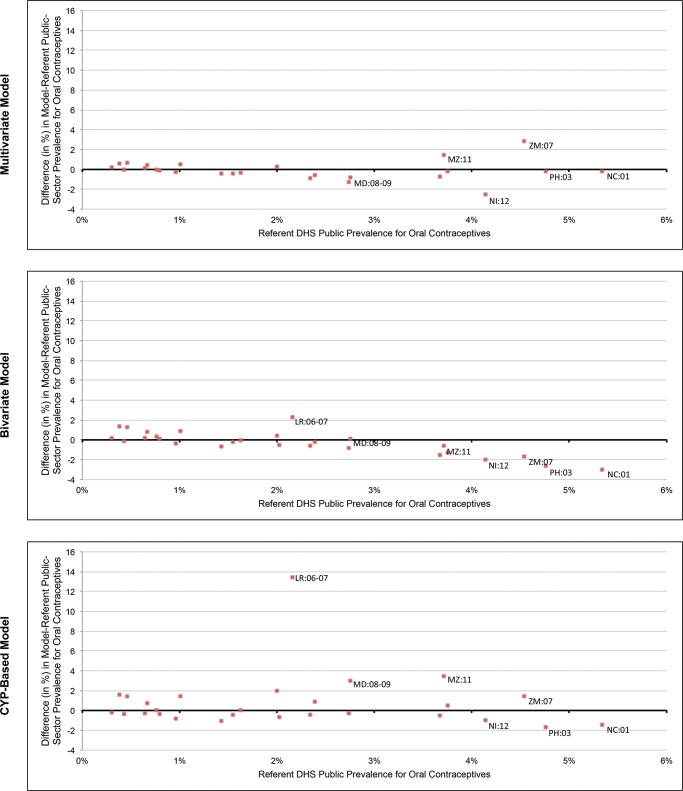
Difference Between Model-Generated and Referent DHS Public Oral Contraceptives Prevalence Rate

**APPENDIX FIGURE 3.**
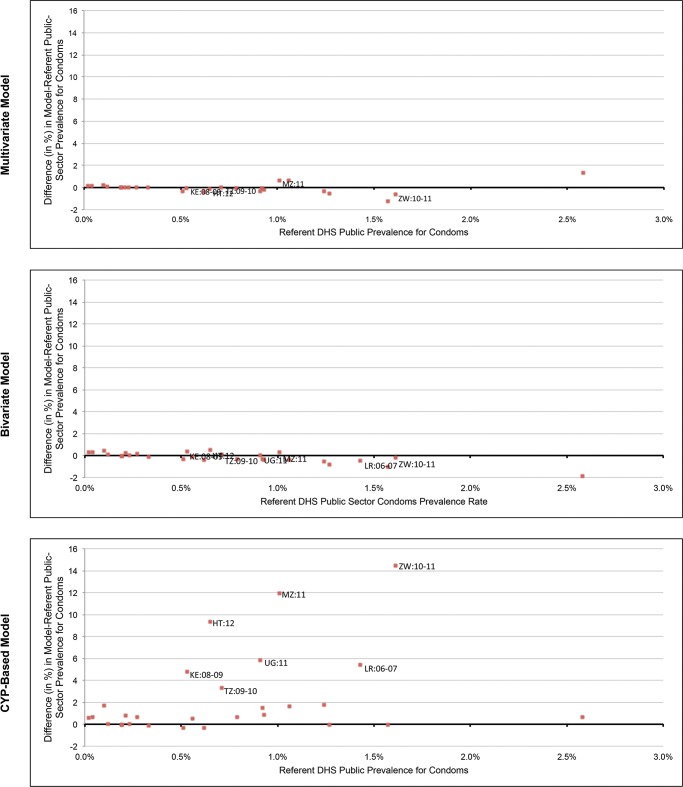
Difference Between Model-Generated and Referent DHS Public Condoms Prevalence Rate

The “error” (i.e., the difference between the model-generated values and the DHS values) for each country and for each model, as well as patterns of error, compared with the referent values can be seen in the appendix figures at the end of this article (Appendix Figure 1, Appendix Figure 2, Appendix Figure 3).

For most models, the error terms are scattered around zero throughout the x-axis, indicating that they are not systematically biased. As previously noted, however, the bivariate models for oral contraceptives (Appendix Figure 2) and condoms (Appendix Figure 3) tend to underestimate the prevalence rates for countries with higher public-sector prevalence values and overestimate prevalence rates for those with lower public-sector prevalence values, while the condoms CYP model tends to systematically overestimate prevalence rates (Appendix Figure 3).

## DISCUSSION

CPR is a vital indicator needed by country governments, international donors, and other stakeholders for measuring national and subnational progress against global initiatives, such as FP2020, Scaling Up Nutrition, and Every Woman Every Child, and for gauging health outcomes. These development partners need robust methods for estimating evolving CPRs, which population-based surveys cannot always provide due to their costs and limited frequencies. Timely and disaggregated CPR estimates thus require high-quality data that are routinely collected and reported.

Our results show a close correlation between the logistics distribution data being collected and actual use of family planning methods, demonstrating the quality of the current logistics data provided through the PPMR and through PipeLine. These results point to the effectiveness of health systems strengthening activities that have focused on strengthening national supply chains and improving data visibility for supply chain management. The results also point to the valuable role that accurate logistics data can play in estimating prevalence of short-acting methods in the interim between nationally representative surveys to help countries monitor their performance and track their progress.

All models, with the exception of the CYP-based model for condoms, were able to estimate public-sector prevalence of short-acting methods to within 2 percentage points in at least 85% of countries. For tracking the general picture of contraceptive prevalence in a country, the potential 2 percentage-point error may provide enough accuracy for planning or for estimating progress in years between surveys. On average, all models except the condoms CYP model performed well, but the regression models were more accurate.

All models, except the CYP condoms model, estimated public-sector prevalence of short-acting methods to within 2 percentage points in at least 85% of countries.

While all 3 models estimate public-sector prevalence of short-acting methods, some differences exist in terms of complexity and accuracy of the models. The CYP-based model offers the simplest calculation method. It estimates contraceptive prevalence based on existing CYP conversion factors, so its interpretation is straightforward—increases in commodity dispensed correlate directly with increases in contraceptive use for the method. No statistical tools are needed, and the only data required are logistics (distribution) and population data. The bivariate and multivariate regression models, although more accurate for a greater number of countries, are also more complex; they were created using regression techniques following a natural log transformation of both the logistics (AMD or AQD) and DHS prevalence data for the method. While being the most consistently accurate, the multivariate regression model also requires a previous prevalence estimate for the method. Further, in the multivariate models, the strength of the relationship between AMD/100 WRA and the referent prevalence rate decreased for oral contraceptives and ceased to be significant for condoms, indicating that one of the best predictors for current use of oral contraceptives or condoms might be previous use of orals or condoms, respectively. In contrast, the relationship for injectable contraceptives remained significant (*P*<.001). When we combine this information with the fact that the condom bivariate and condom CYP models over- or underestimate prevalence rates differentially, we note that estimating condom prevalence rates using logistics data is problematic. For detailed program planning purposes, when high accuracy is more important, the bivariate and multivariate regression models provide slightly more accurate estimates on average—in our analysis their average and maximum error values were smaller.

The CYP model offers the simplest calculation method while the regression models were more accurate.

Country-specific factors that would directly affect the relationship between logistics data and contraceptive use data are not captured in our analysis. These include differences in our logistics data sources (forecasted dispensed-to-user; versus issues data, which tend to be higher than dispensed-to-user data; versus actual dispensed-to-user data) and possible differences in wastage rates in individual countries. Much of our data represented either forecasted dispensed-to-user data or movements of stock from central levels to peripheral levels rather than actual data on contraceptives dispensed to clients. Given the more direct link between commodities dispensed to users and commodities used (as opposed to commodities issued at a higher level and those used by clients), we would expect greater accuracy and precision from each model as donors and country governments continue to strengthen their LMISs and actual dispensed-to-user data become more available.

The strengths of the results of our models vary between contraceptive methods, indicating some inherent limitations in using logistics data to estimate prevalence rates. Contraceptives such as condoms (especially) and oral contraceptives dispensed by health facilities may not be used immediately (or completely) by clients. In addition, linking logistics data for condoms, which have a dual purpose of preventing sexually transmitted infections (STIs) and unintended pregnancy, with family planning survey data is complicated. Women may not report their use of condoms for contraception during a DHS survey because they associate the condoms more with STI prevention, they associate condoms as a method that their partner uses (not one that the women themselves use), or they might report using another more effective contraceptive method, in which case that method is recorded rather than condoms.^25^^-^^27^ Additionally, condoms dispensed from STI clinics may be used for family planning but may not be captured in the logistics data. In comparison, oral and injectable contraceptives have a single use, and thus the link between dispensing and intended use is stronger. This may help explain why the results for condoms are not significant for the multivariate model and why the higher error terms in the condoms CYP model for several countries with high HIV prevalence were seen (Appendix Figure 3).

Some outliers may also be explained by situations in which methods are provided by the public sector to NGO or private outlets; in these cases, clients would have reported receiving these products outside the public sector, and this use would not be captured by the DHS public-sector prevalence values. However, in some countries, PipeLine and PPMR capture these products along with the products destined for public-sector consumption. Conversely, in countries with weaker or rebounding public systems, NGOs may provide products to public-sector facilities, and therefore the logistics data are not captured in PipeLine or PPMR.

The accuracy of our models and similar research in Rwanda at the district level strongly suggest that these models could also be used to help countries evaluate district-level contraceptive prevalence for improved in-country targeting of family planning resources.^12^ Current tools for estimating CPRs, namely national population-based surveys, are rarely representative at such disaggregate scales due to sampling constraints.

Countries could use routine logistics data with these models to evaluate district-level contraceptive prevalence, and thus better target family planning resources.

Lacking private-sector logistics data, we focused on public-sector logistics data and CPRs, and thus we were unable to estimate overall country prevalence rates for short-acting methods. In principle, the same strategy for developing the models should be possible with private-sector logistics data where they are available. In the countries for which we had data, the average public-sector market share was 53% for oral contraceptives and 78% for injectable contraceptives, while for male condoms it was 24%. In countries with high public-sector market share, contraceptive prevalence estimates and trends from public-sector logistics data will be more reflective of the overall contraceptive prevalence than in countries where the public-sector market share is low. While in all cases, we recommend seeking both public- and private-sector data, the latter are even more critical for condoms given the higher private-sector market share.

Additionally, when evaluating the models, we are comparing the results against DHS-based estimates, which (although considered the current gold standard for most demographic and health indicators) have their own limitations.^28^ The standard errors for statistics related to contraceptive method use and public-sector source of supply vary by survey (typically calculated in the range of 2–3 percentage points), and results are calculated to fall within a ±2 times the standard error of that statistic in 95% of cases. Additionally, results are based on lengthy interviews, and responses might be biased or misinformed. For example, social marketing products can be provided at public-sector facilities directly by health workers or in a kiosk within the public facility. During the survey, clients may not differentiate between the public versus social marketing sources and may report the public sector as the source of these social marketing products.

### Future Research

As with similar models, adding more data is likely to strengthen their predictive power. A greater availability of actual dispensed-to-user data and of country-specific wastage rates would improve the reliability of using logistics data to estimate prevalence rates by method.

The CYP model consistently overestimated public-sector condom use levels, indicating a possible need for further research reexamining the existing conversion factors and the DHS condom use values in more detail. Future research should focus on country-specific factors that might be used to adjust CYP conversion factors or produce country- or region-specific conversion factors in order to improve the accuracy of the model for condoms. Data permitting, future research should also include total condom use in the regression models instead of condom use specifically for contraceptive purposes.

Due to the limited number of countries (n = 30) for which we had overlapping DHS and logistics data, we included only one additional variable in the multivariate model. As the number of countries with both logistics data and population survey-based prevalence data by method expands, future research could include additional variables such as demand generation initiatives, product costs, product flow between sectors, consistency of product availability, gross national income per capita, HIV prevalence, and average education level. These factors would be especially relevant for cases where the logistics data represent issues data, rather than dispensed-to-user data, as most of these covariates would influence whether or not clients accessed services at the facility level.

Additionally, while short-acting methods currently dominate the method mix in most countries, as the international community focuses on expanding long-acting method options, we anticipate that their contributions to the CPR will increase substantially. Due to lack of a sufficient quantity of overlapping logistics and DHS data, we were unable to adequately construct models for long-acting contraceptive methods. As more data become available, it will be important to develop models for these long-acting methods.

Further research should also explore expanding the models to estimate prevalence rates beyond the public sector, through the addition of data from nonpublic sources, when available, or by adding market share data along with the public-sector logistics data. With sufficient data, the models could be expanded to estimate total CPR, allowing countries to track their progress against national and global family planning goals.

### Limitations

We selected countries for this study on the basis of overlapping DHS and logistics data availability. As a result, the number of data points included in each model is quite small (n≤30). This affected our analysis in 2 ways. First, the selection criteria may have introduced bias into the results if countries that have better data availability have stronger associations between prevalence rates by method and contraceptive distribution. Second, with the limited data points, we were unable to create separate datasets for model building and model validation. Countries that were not included in the analysis may behave differently than those that were included. While we constructed our models based on the best available data, testing it on countries not included in the model construction would provide us with a better evaluation of the models’ external validity.

Additionally, because the models use data from many countries to estimate results for a particular country, potential for variation exists where the relationship between logistics data and the prevalence rate within a country is stronger or weaker than the average of the countries used in creating the model.

For 2 countries, Bangladesh and Jordan, the DHS included only ever-married women as respondents for the women’s questionnaire. While the majority of WRA in both of these countries were currently (80.4% in Bangladesh) or previously in union (54.4% in Jordan) at the time of the surveys, patterns of contraceptive use among these women might differ substantially from women who had never been married. Consequently, the model results would have been affected. A final limitation of the models, which estimate future prevalence rate of short-acting methods from the past relationship of the logistics data and the DHS prevalence rate data, is that they rely on an implicit assumption that the relationship between logistics data and client use remains constant over time. As countries improve the quality of their logistics data, and as more countries begin collecting information on contraceptives dispensed to users rather than contraceptives issued from warehouses, the models may need to be recalibrated.

## CONCLUSIONS

With less than 5 years remaining to meet FP2020 goals, national family planning program managers and international donors need frequently updated data on current CPRs in order to most effectively target limited program resources. Demonstrating the strength of the relationship between logistics data and prevalence estimates for short-acting methods is an important first step in showing the potential of using logistics data to provide a low-cost alternative for generating routine CPR estimates. Our results show a strong relationship between public-sector contraceptive logistics data and public-sector prevalence rates for short-acting methods, demonstrating the quality of current logistics data and their ability to provide relatively accurate prevalence estimates. Using logistics data for estimating condom use levels, however, should be done with caution given the relative weakness and limitations of the condoms models. Future work relating subnational logistics data with CPR, and tracking that relationship over time, is needed; there is also a need for expanding the models to estimate prevalence rates by method beyond the public sector through the addition of data from nonpublic sources, when available, or by adding market share data along with the public-sector logistics data.

Our models demonstrate strong, significant relationships between public-sector contraceptive logistics data and referent public-sector prevalence rates for short-acting methods.

As the international community continues to work to improve the health of women and children, investments in health information systems and supply chains will be essential to meet the challenges to improve access to contraceptives and reduce unmet need for family planning. It will be equally important for family planning and supply chain program managers to work together to share and effectively use these data.

Depending on the data available, the level of precision sought, and the importance of being able to describe the model used to a general audience, national and international stakeholders can use any of the 3 models to estimate country-level prevalence rates of short-acting methods at times when timely estimates from nationally representative survey data are not available. These models provide a starting point for generating these interim estimates. Given the additional complexities of the regression-based analysis, we recommend use of the CYP-based model (accurate to within 2 percentage points for most countries) for estimating national prevalence rates by method. The results from these models should be triangulated against other available data to allow stakeholders to best prioritize family planning and supply chain interventions.

## Notes

**Peer Reviewed**
